# Development and validation of a nomogram for predicting low birth weight among pregnant women who had antenatal care visits at Debre Markos Comprehensive and Specialized Hospital, Ethiopia

**DOI:** 10.3389/fmed.2023.1253490

**Published:** 2023-11-28

**Authors:** Bezawit Melak Fente, Getayeneh Antehunegn Tesema, Temesgen Worku Gudayu, Mengstu Melkamu Asaye

**Affiliations:** ^1^Department of General Midwifery, School of Midwifery, College of Medicine and Health Sciences, University of Gondar, Gondar, Ethiopia; ^2^Department of Epidemiology and Biostatistics, Institute of Public Health, College of Medicine and Health Sciences, University of Gondar, Gondar, Ethiopia; ^3^Department of Clinical Midwifery, School of Midwifery, College of Medicine and Health Sciences, University of Gondar, Gondar, Ethiopia; ^4^Department of Women's and Family Health, School of Midwifery, College of Medicine and Health Sciences, University of Gondar, Gondar, Ethiopia

**Keywords:** Ethiopia, pregnant women, nomogram, low birth weight, prediction model

## Abstract

**Background:**

Birth weight is a crucial factor linked to a newborn’s survival and can also affect their future health, growth, and development. Earlier, researchers focused on exploring maternal and fetal factors contributing to low birth weight. However, in recent years, there has been a shift toward effectively predicting low birth weight by utilizing a combination of variables. This study aims to develop and validate a nomogram for predicting low birth weight in Ethiopia.

**Methods:**

A retrospective follow-up study was conducted, and a total of 1,120 pregnant women were included. Client charts were selected using a simple random sampling technique. Data were extracted using a structured checklist prepared on the KoboToolbox (Cambridge, Massachusetts in the United States) and exported to STATA version 14 (Computing Resource Center in California) and R version 4.2.2 (University of Auckland, New Zealand) for data management and analysis. A nomogram was developed based on a binary logistic model, and its performance was assessed by discrimination power and calibration. Internal validation was performed using bootstrapping. To evaluate the clinical impact, decision curve analysis was applied.

**Results:**

The nomogram included gestational age, hemoglobin, primigravida, unplanned pregnancy, and preeclampsia. The AUROC of the predicted nomogram was 84.3%, and internal validation was 80.1%. The calibration plot indicated that the nomogram was well calibrated. The model was found to have clinical benefit.

**Conclusion:**

The nomogram demonstrates strong discrimination performance and can predict low birth weight clinically. As a result, it can be used in clinical practice, which will help clinicians in making quick and personalized predictions simply and rapidly, enabling the early identification and medical intervention. For broader applicability, the nomogram must be externally validated.

## Introduction

Birth weight is a reliable indicator of fetal development and might have a significant role in predicting the newborn’s survival and can also affect their long-term health, growth, and development (1). Low birth weight (LBW), defined by World Health Organization (WHO) standards, refers to birth weight lower than 2,500 g, while the normal infant weight ranges from 2,500 g to 4,000 g (1). Babies with low birth weight are born as a result of prematurity (being born too soon), poor intrauterine growth (developing too slowly in the womb), or both (2, 3). Approximately 20 million babies with low birth weight are born each year, making it a persistently significant global public health problem (1). It is noteworthy that the majority of these babies are born in developing countries (4). The prevalence of LBW varies widely across different regions and nations, with highest incidence often found in low- and middle-income countries (LMIC). The prevalence in low- and middle-income nations (16.5%) is more than twice as high as that in high-income countries (7%) (4, 5). The LBW rate in Ethiopia ranges from 8 to 54%, with large variations across geographical contexts and time periods. A recent systematic analysis found a pooled estimate of 18% in Ethiopia (6), implying that it is still a significant public health issue in the country.

Globally, 60–80% of neonatal mortality is caused by LBW (7). Infant morbidity and mortality rates are higher in cases where the newborn has LBW or is small for gestational age (8). It is associated with adverse effects on a child’s health, such as decreased cognitive function, increased risk of infection, neurological abnormalities, hypertension, type 2 diabetes, and later cardiovascular diseases (2, 6, 9–15). Infants with low birth weight have a mortality rate that is nearly 20 times higher than infants with a normal weight (1). A major amount of Ethiopia’s infant mortality rate, recorded at 48 deaths per 1,000 live births, was associated with LBW (16).

Maternal age, birth order, household income, primigravida, educational level, rural residence, diet, anemia, parity, presence of chronic illness, maternal nutritional status before and throughout pregnancy, preeclampsia, short stature, sex of the newborn, physical labor during pregnancy, and antenatal care (ANC) are well recognized risk factors for LBW (4, 6, 13, 15, 17).

The most effective strategy for preventing LBW is to initiate prenatal care as early as possible in pregnancy and to continue receiving it throughout pregnancy (8). By the end of 2025, the World Health Assembly set a strategic aim to reduce LBW by 30%(from approximately 20 million to nearly 14 million infants with low weight at birth) (1). The care provided during the prenatal, antenatal, intranatal, and postnatal periods, interventions to prevent LBW, and LBW-associated morbidity and mortality in community settings are emphasized in the packages (7). The prediction model is used to identify pregnant women who are at high risk of having babies with LBW early on, to make informed clinical decision-making, and to improve the excellence of early attention to lower morbidity and death rates for newborns and children under the age of five. Therefore, developing an easy, non-invasive, useful, and truthful model for determining the risk of LBW in different periods of pregnancy is crucial, particularly in resource-limiting settings where there is a shortage of imaging equipment and trained personnel to diagnose or predict fetal growth restrictions earlier in the gestation period. Hence, the objective of this study is to develop and validate a nomogram for LBW in the context of Ethiopia.

## Methods and materials

### Study design

A retrospective follow-up study was conducted at Debre Markos Comprehensive and Specialized Hospital (DMCSH) in Ethiopia to develop and validate a nomogram for LBW among pregnant women who had antenatal care visits.

The study’s hypothetical design was proposed as follows: the incidence of LBW at a future time “*t*” is a function of various prognostic determinants measured at a time point before the occurrence of LBW or during pregnancy, “*t*0.” The study’s domain included pregnant women who received antenatal care.


(PTB(t0+1)=f{x1(t0)+x2(t0)+x3(t0)+…})

### Study period, area, and population

The study was conducted at DMCSH from 1 January 2020 to 30 August 2022, and the data were extracted from 3 January 2023 to 1 February 2023. DMCSH is located at Debre Markos town, which is 299 km from Addis Ababa, Ethiopia’s capital city and 265 km from Bahir-Dar, the capital city of the Amhara Regional State. This hospital is one of the largest tertiary-level referral facilities in the Amhara region. The history of this hospital dates back to 1965 when it was founded by Emperor H/Selassie, and currently, it serves approximately five million people (18). Gynecology and Obstetrics is one of the major departments in the hospital. Antenatal care and delivery services are among the services provided in the hospital. Since last year, the total number of pregnant women receiving antenatal care and the total number of deliveries have been 2,200 and 6,000, respectively. The hospital has 7 obstetrics and gynecology specialists, 46 midwives, 1 clinical midwifery specialist, and 3 emergency surgeons. The study population was all pregnant women who had ANC visits at DMCSH from 1 January 2020 to 30 August 2022. At DMCSH, all pregnant women who received ANC follow-ups from 1 January 2020 to 30 August 2022 and gave birth at DMCSH were included.

### Sample size and sampling procedure

A rule of thumb for developing a prediction model suggests having 10 to 20 events for each predictor (19). Generally, the required sample size in the development of a prediction model is at least 10 events for each predictor (19). The formula for determining the required sample size is *N* = (n*10)/*I*, where n represents the number of predictors (19). In this study, considering 15 predictors with easily accessible and significant clinical or statistical effects for low birth weight, the incidence of LBW was 14.0% (13), and the calculated sample size can be derived as follows: *N* = (n*10)/*I*, (15*10)/0.140 = 1,071. Accounting for a 5% for missing data, the adjusted sample size was 1,124. Using a computer-generated simple random sampling procedure, 1,124 records (charts) of study participants were selected.

### Variables of the study

#### Dependent variable

Low birth weight (yes/no).

#### Independent variables

Age, marital status, middle upper arm circumference (MUAC), maternal weight, place of residence, gravidity, hemoglobin level, pregnancy status, timing of ANC initiation, timing of iron folate initiation, comorbidities (such as pulmonary: history of asthma or COPD (chronic obstructive pulmonary disease); cardiac: history of heart failure or ischemic heart disease; and renal diseases), and preeclampsia.

### Operational definitions

#### Anemia

Mothers with hemoglobin level less than 11 g/dL (1).

#### Gravidity

The total number of pregnancies, including abortion, ectopic pregnancy, and any other pregnancies documented on the chart.

#### Low birth weight

Any neonate that weighs less than 2,500 g (5.51 pounds). Within 72 h of delivery, the birth weight was measured on a digital scale (1).

#### Late initiation of antenatal care

Pregnant women who start antenatal care visit after 12 weeks of gestation (20).

#### Under nutrition or malnourished

Mother with MUAC less than 24 cm during pregnancy (1).

#### Parity

The number of deliveries after 28 weeks of gestation including IUFD and stillbirth documented in the chart.

#### Underweight

Mothers with weight less than 50 kg (9).

### Data collection procedure and tool

A data extraction checklist was prepared on the KoboToolbox web-based tool for the collection of data from the mother’s medical records (21). The checklist was arranged into sociodemographic characteristics of the pregnant mothers, medical illnesses, past and recent obstetric characteristics, and birth outcomes.

### Data quality control and assurance

Data extraction checklist was developed in English for data collection. Prior to the actual data collection, the primary investigator trained the data collectors for 2 days about the KoboToolbox, the elements of the checklist, the sequence, and potential issues they might encounter. The supervision of data collectors was done often and on schedule. The principal investigator verified the correctness and completeness of the data during data collection.

### Data processing and analysis

Data were collected using KoboToolbox. The collected data were exported to STATA 14 and R 4.2.2 Software for data management and analysis. Missing data were handled by multiple imputation by assuming missing values at random. The Transparent Reporting of a multivariable Prediction model for Individual Prognosis or Diagnosis (TRIPOD) guideline was used for developing and reporting the prediction model (22). Tables and figures are used to describe the characteristics of the study participants.

A logistic regression analysis was used to evaluate which variables are most powerful in predicting LBW (23). A bivariable regression analysis was used to obtain insights into the association of each potential determinant with low birth weight and for inclusion in a multivariable regression analysis. Variables with a *p*-value of ≤0.25 in the bivariable analysis were fitted to the multivariable regression analysis. After a stepwise backward elimination technique was used, the role of each predictor in the multivariable analysis was assessed by the likelihood ratio test. To be more liberal, a *p*-value ≤0.15 for the likelihood ratio test was used to fit the reduced model (23).

**Figure 1:**
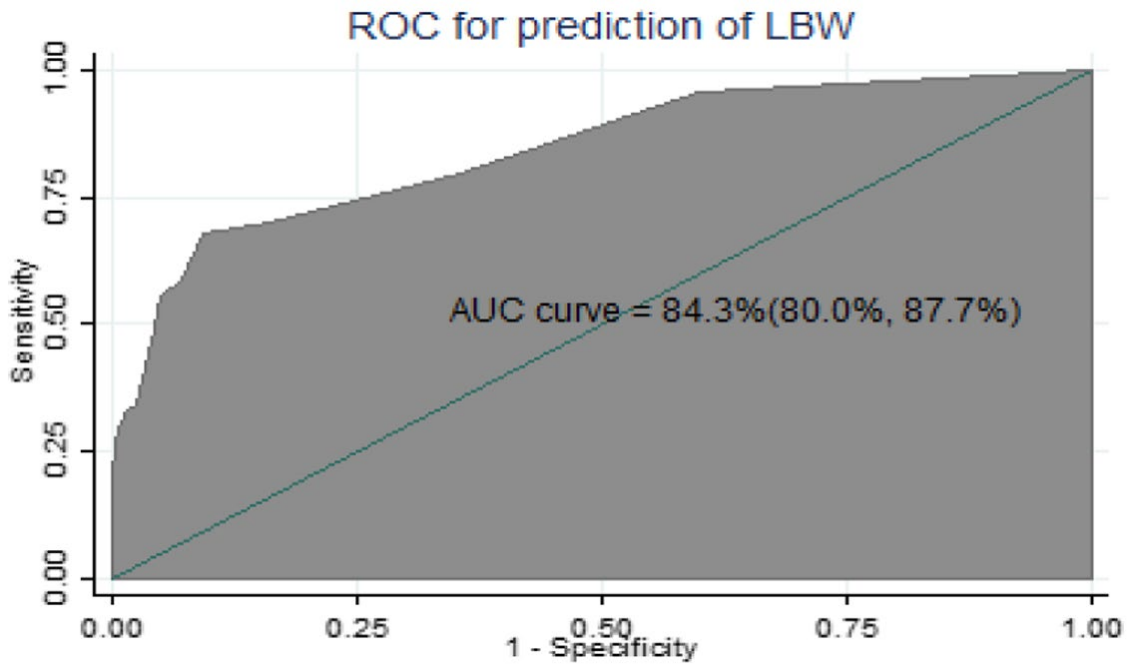
Calibration plot for the developed model.

The performance of the nomogram was assessed using discrimination power and calibration. Discrimination refers to the performance of the model to differentiate pregnant women who give birth to a LBW baby from those who did not give birth to a LBW baby. The area under the receiver operating characteristic curve (AUROC) was used to evaluate the discrimination power of nomogram. The AUROC between 0.7 and 0.9 indicates good discrimination power and > 0.9 indicates excellent discrimination power (23, 24). Model calibration is the agreement between the observed proportions of preterm birth and predicted probabilities of LBW (24). Calibration was assessed using a calibration plot and a value of p to ensure the reliability of the prediction model, and Figure 1 shows that the model is well calibrated calibration, with the calibration plot falling along a 45° line or an insignificant statistical test, such as the Hosmer-Lemshow test (24). Internal validation was performed using the bootstrap procedure, which replicated the sample 1,000 times, to estimate how successfully the nomogram developed on the development set would perform on a hypothetical set of new patients (23). This approach can particularly assess the stability of selected predictors, as well as prediction quality (24). The most effective cutoff point for classifying pregnant women’s risk of having LBW babies as high or low was determined using the Youden index (25). To evaluate the prediction effectiveness, sensitivity, specificity, positive likelihood ratio, negative likelihood ratio, and accuracy were utilized. The clinical benefit of the nomogram was evaluated using decision curve analysis (DCA) (26). DCA was used to indicate the balance between the harm of a false-positive classification and the benefit of a true-positive classification (26). To provide a simply clinically applicable, individual prediction of LBW, a nomogram graphical display was developed (27).

## Results

### Baseline demographic, obstetric, and clinical characteristics of pregnant women

We included 1,120 women who gave birth and had their birth weight taken within 72 h of delivery. The demographic, obstetric, and clinical features of pregnant women enrolled in the study are shown in Table 1. The mothers’ median age was 28 years (IQR: 25–30), while 48 (4.3%) of the participants were under the age of 20. The majority of them were married. More than one-third (36.8%) were primigravid, and a quarter (25%) of the mothers initiated an ANC visit early in their pregnancy. Unplanned pregnancies accounted for one-third (33.4%) of all pregnancies. Of the 1,120 women, 24 participants (2.14%) have a past medical history of chronic co-morbidities, which could include cardiovascular, pulmonary, diabetic, or chronic renal illness. According to the hemoglobin test results, 49 (4.3%) participants had hemoglobin levels less than 11 g/dL. Of the 1,120 women, 137 (12.23%) had preeclampsia (Table 1).

**Table 1:** Demographic, obstetric, and clinical characteristics of pregnant women included in the analysis.

Predictor	Category	Frequency	Percent
Gravidity	Primigravida	412	36.79
	Multigravida	708	63.21
Preeclampsia	No	983	87.77
	Yes	137	12.23
Residence	Urban	975	87.05
	Rural	145	12.95
Weight	<50 kg	193	17.23
	> = 50 kg	927	82.77
MUAC	> = 24 cm	853	76.16
	<24 cm	267	23.84
Hemoglobin	> = 11 g/dL	1,071	95.63
	<11 g/dL	49	4.38
Timing of iron folate initiation	1st trimester	262	23.39
	2nd trimester	826	73.75
	3ed trimester	32	2.86
Pregnancy status	Planned	745	66.52
	Unplanned	375	33.48
Timing of ANC initiation	Early initiation	280	25.00
	Late initiation	840	75.00
Age	> = 20	1,072	95.71
	<20	48	4.29
Marital status	Married	1,099	98.13
	Single	21	1.88
Rh status	Positive	1,040	92.86
	Negative	80	7.14
HIV	Negative	1,067	95.27
	Positive	53	4.73
Comorbidities	No	1,096	97.86
	Yes	24	2.14

MUAC: Middle Upper Arm Circumference.

### Prediction model for low birth weight

#### Variable selection

Out of 1,120 women who gave birth, 166 (14.8%) had LBW newborns (SD: 516.1). The average birth weight was 2,942.7 g. Following a study of the literature, 14 baseline measurements on the mother’s demographic, obstetric, and clinical features were taken into consideration to predict LBW. Numerous factors were determined to be eligible for inclusion in the prediction model using the univariable analysis. Age at current pregnancy, hemoglobin level, gravidity, marital status, residency, weight, MUAC, timing of ANC initiation, pregnancy status, and preeclampsia were the variables with a *p*-value ≤0.25 in the univariable analysis. Using the results, a prediction model was developed, and the equation for the prediction model was obtained (Table 2).

**Table 2:** Univariable and multivariable logistic regression analyzes of predictors of low birth weight among pregnant women who had ANC visit to predict low birth weight (*n* = 1,120).

Predictor variable	Univariable analysis		Multivariable analysis	
	β (95% CIs)	p-value	β (95% CIs)	p-value
Age (<20)	2.780 (2.149, 3.424)	<0.001^	2.605 (1.800, 3.410)	<0.001*
Residency (rural)	0.945 (0.532, 1.358)	0.009^	0.325 (−0.234, 0.88)	0.254
Marital status (single)	0.890 (−0.071, 1.85)	0.070^	−0.168 (−1.61, 1.28)	0.820
Gravidity (primigravida)	0.387 (0.050, 0.725)	0.025^	0.423 (−0.921, 0.07)	0.096*
Comorbidities (yes)	0.461 (−0.539, 1.46)	0.366	NA	
HIV (positive)	0.286 (0.580, 1.153)	0.517	NA	
Preeclampsia (yes)	2.535 (2.132, 2.938)	<0.001^	2.647 (2.169, 3.126)	<0.001*
Rh (negative)	0.056 (−0.715, 0.604)	0.869	NA	
Weight (<50 kg)	0.555 (0.158, 0.952)	<0.001^	−0.054 (−0.704, 0.59)	0.870
MUAC (< 24 cm)	0.819 (0.464, 1.175)	0.080^	0.287 (−0.282, 0.85)	0.324
Hemoglobin (11 g/dL)	3.383 (2.690, 4.075)	<0.001^	3.336 (2.528, 4.143)	<0.001*
Timing of iron folate initiation	0.824 (0.404, 2.052)	0.388	NA	
Timing of ANC initiation (Late)	0.297 (−0.112, 0.706)	0.155^	−0.001 (−0.509, 0.50)	0.998
Pregnancy status (unplanned)	1.098 (0.757, 1.438)	<0.001^	0.597 (0.145, 1.049)	0.010*

*Variables significantly associated to LBW used for model development, ^Variables of a *p*-value ≤0.25 required multivariable analysis, NA: not applicable and the actual observed value.

#### Model development

Probability of LBW = f (predictor variables).

PR (LBW) = f (age, pregnancy status, gravidity, preeclampsia, hemoglobin).

PR (Y = 1) = f (X), where Y = 1 refers to having LBW and Y = 0 refers to not having LBW.


$$\left(PR\left(Y=1\right)=f\left\{\begin{array}{l} \beta o+\beta 1\;age+\beta 2gravidity+\beta 3\;pregnancy\;status\\ +\;\;\beta 4+preeclampsia+\beta 5\;HGB \end{array}\right\}\right)$$

$$\begin{array}{l} \mathrm{Risk prediction model}=2.78\;\mathrm{Age}\;\left(<20\right)\\ +\;1.098\;\mathrm{Pregnancy status}\;\left(\mathrm{unplanned}\right)+3.383\;\mathrm{Hemoglobin}\;\left(11g/\mathrm{dL}\right)\\ +\;0.387\;\mathrm{Gravidity}\;\left(\mathrm{primigravida}\right)+2.535\;\mathrm{preeclampsia}\;\left(\mathrm{Yes}\right). \end{array}$$

#### Model performance

**Figure 2:**
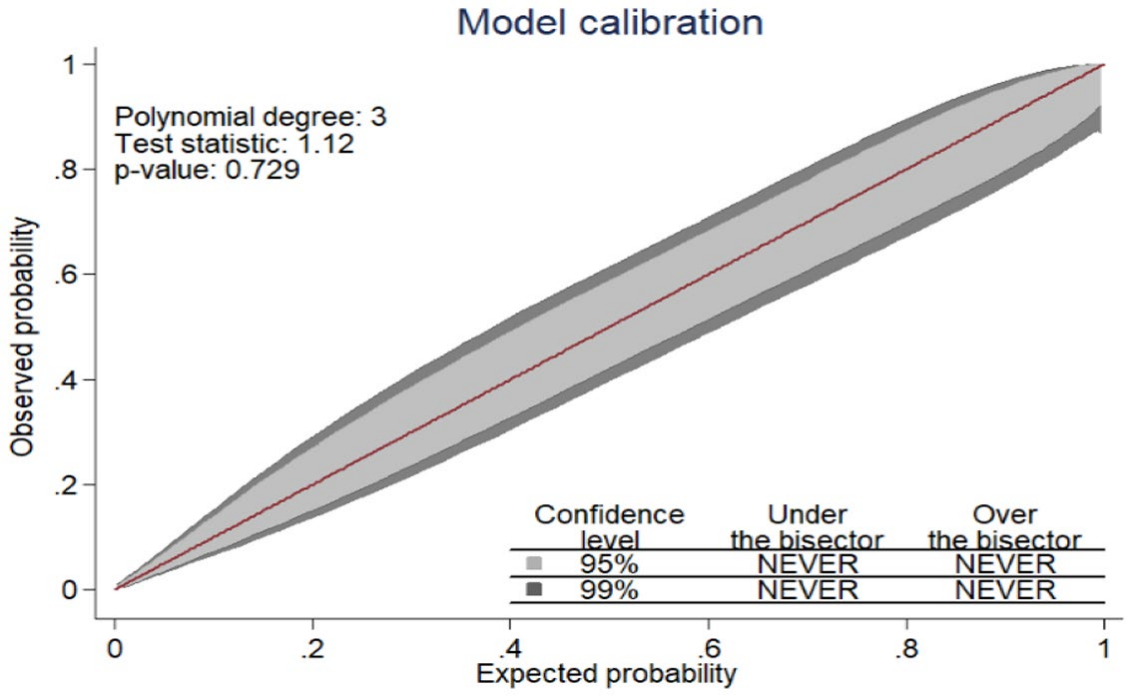
The ROC curve represents the probability of risk for LBW among pregnant women who had ANC visits.

The AUROC of the nomogram using five predictors was 84.3% (95% CIs: 80.0, 87.7%) using original beta coefficients, which means a model that was 84.3% differentiates pregnant women who give birth to LBW from those who do not give birth to LBW, indicating the model’s capacity for identification was good (Figure 2).

Since the model was well calibrated (*p* = 0.729) and the calibration plot fell along a 45° line (Figure 1) or an insignificant statistical test, such as the Hosmer–Lemshow test (*p*-value = 0.066). it indicates that the model well represented the data (the nomogram showed probability, and its actual probability was in agreement).

### Nomogram for low birth weight

**Figure 3:**
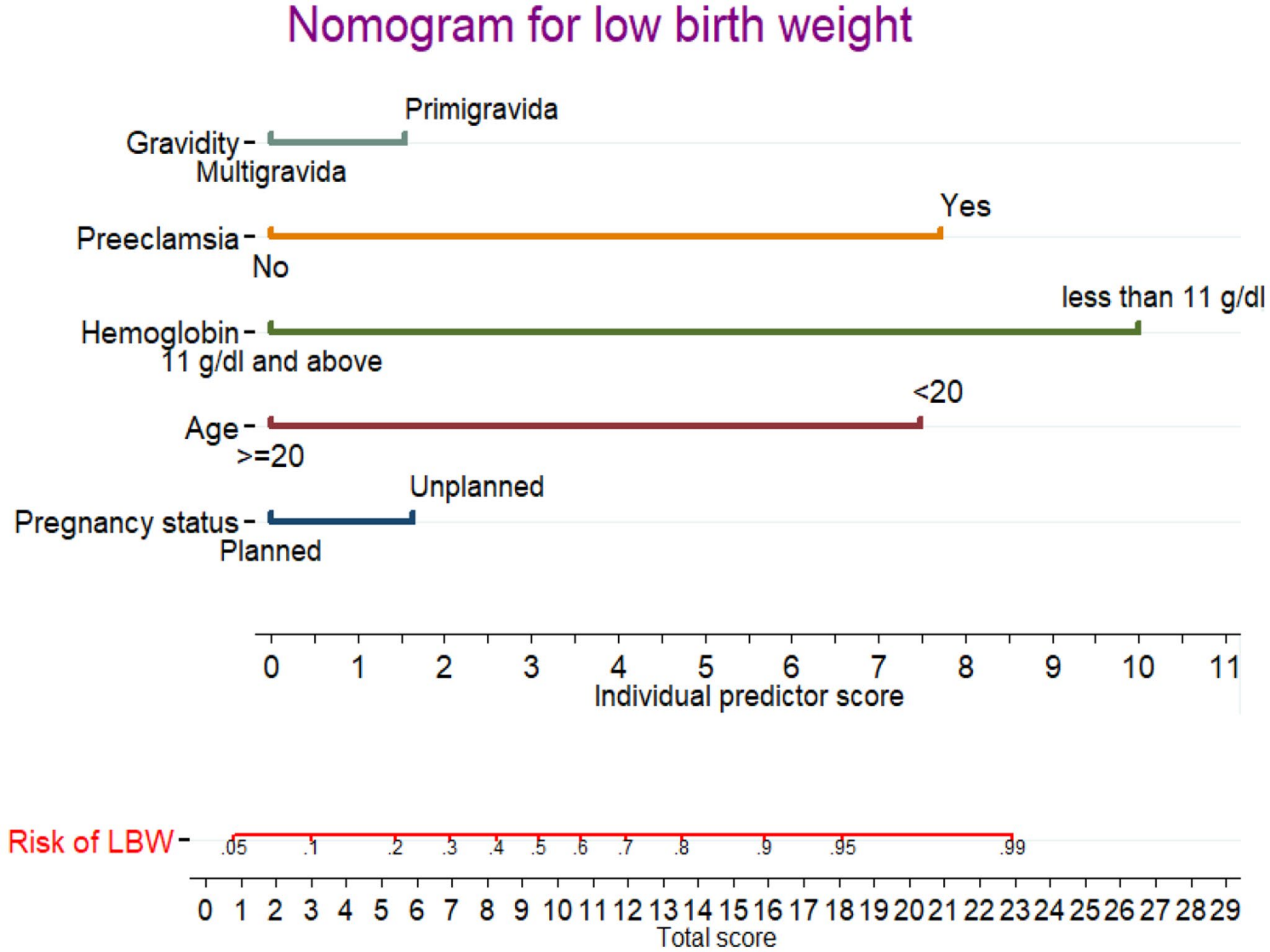
Nomogram for predicting LBW during pregnancy. Instructions: the point score of each risk factor can be calculated separately by reading the score above the factor vertically. Then, the points from each variable value were summed. The sum on the total points scale was located and vertically projected onto the bottom axis, and then, a personalized low birth weight risk was obtained.

A nomogram was developed to generate a simple clinically applicable individualized prediction of pregnant women’s risk of having an LBW baby (Figure 3). Clinicians can calculate the probability of LBW risk in pregnant women by combining each of the values of the prognostic indicators from the nomogram. This graphical representation of a nomogram provides a clinically valuable individualized prediction of LBW risk. Five prognostic markers of LBW were evaluated in the nomogram to predict LBW.

In the nomogram, the linear predictor of five factors was assessed for determining the probability of each pregnant woman to give a LWB baby. For example, for a pregnant women having unplanned pregnancy and primigravida, the anticipated probability of a LBW is as follows: the total point for the two predictors is the sum of the points for each predictor, which is 1.7 + 1.8 = 3.5. As a result, the probability of the LBW was 0.12 (12%), with the equivalent total points in the nomogram showing a low risk of the LBW. For a pregnant women aged <20 and having preeclampsia, the total point was as follow: 7.4 + 7.6 = 15.0 points. Thus, the probability of giving birth to an LBW baby, which is approximately 0.86 (86%), is high.

The best cutoff point value (Youden index) to predict a high or low risk probability of LBW was 0.220 (22.0%). The Youden index cut point value has a maximum sensitivity and specificity of 78.0 and 91.0%, respectively. At the Youden index threshold value, the positive and negative predictive values were 79.4 and 86.1%, respectively. The AUROC curve at the optimal cut point was 80.0%.

#### Validation of the nomogram

**Figure 4:**
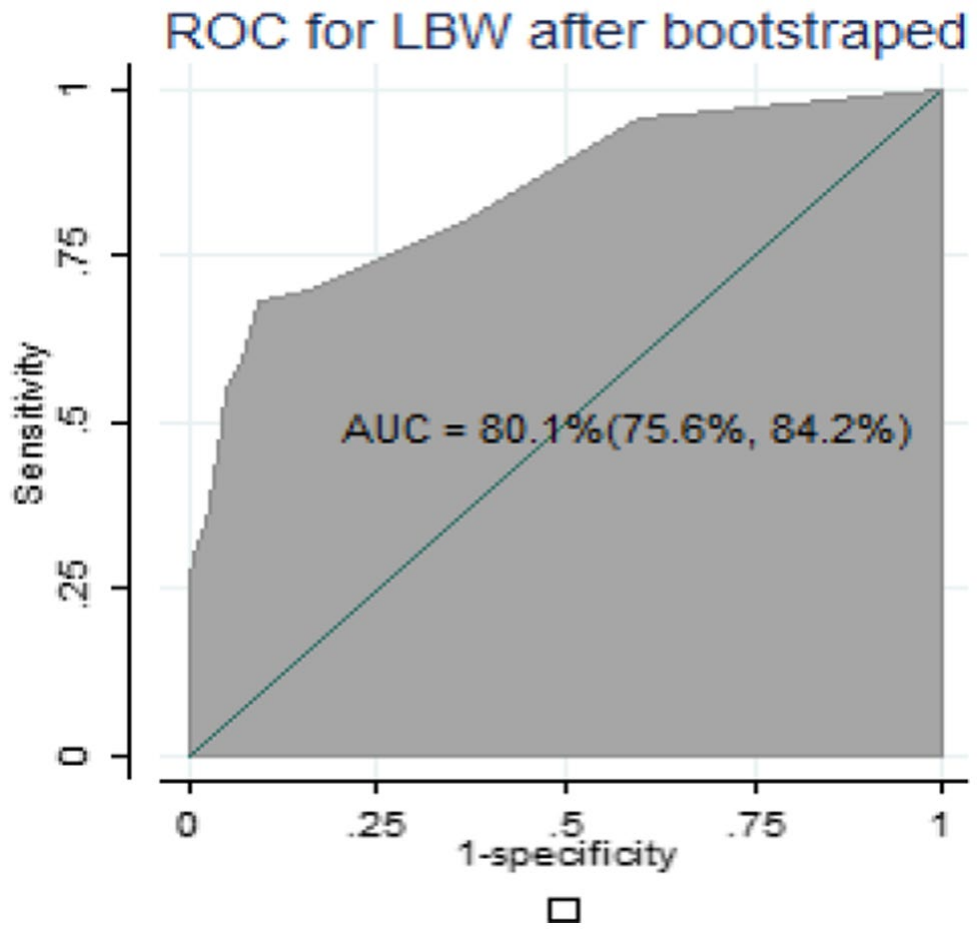
Area under the ROC curve of nomogram after bootstrapping.

For internal validation of the nomogram, 1,000 random bootstrap samples with replacement were generated from the dataset that included complete data on all predictors. After bootstrapping, the predictive performance was 0.801(80.1%) (Figure 4), which is comparable to the performance of the original sample and can be expected when the nomogram is applied to similar future populations.

#### The decision curve analysis (DCA)

**Figure 5:**
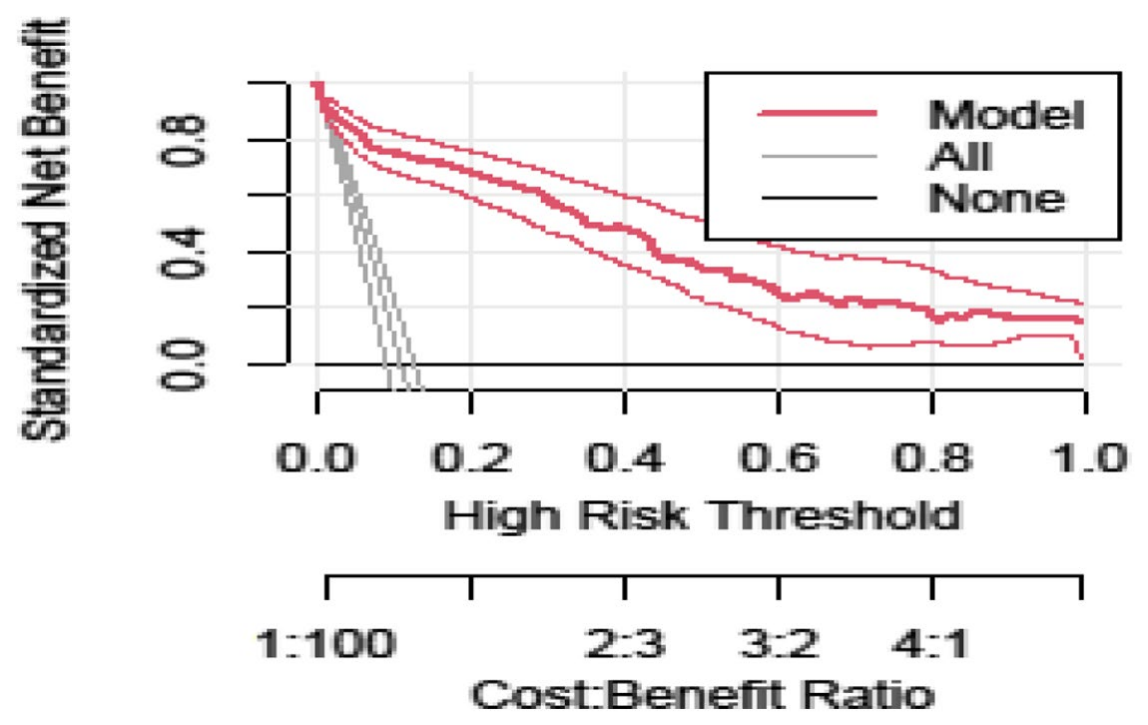
Decision curve analysis curve of the developed model plotted net benefit versus the threshold probability between the model and two extreme scenarios.

Figure 5 shows the LBW nomogram’s decision curve analysis. The decision curve analysis results indicated that individuals with an LBW threshold probability greater than 12% would benefit the greatest from implementing our prediction tool. In general, this study showed that maternal characteristics during pregnancy can be used to predict LBW. The model might be helpful in identifying pregnant women who are at a higher risk of having a baby with LBW. This feasible prediction model would make it possible for the reduction of obstetric-related problems, thus enhancing the overall mother and child healthcare in a low resource setting (Figure 5).

## Discussion

In this study, we constructed a nomogram model based on the optimal combination of maternal characteristics to predict the risk of LBW during pregnancy in areas where no laboratory tests and ultrasound scans are not included. This model includes factors such as age under 20 years, hemoglobin levels below 11 mg/dL, primigravida, unplanned pregnancy, and preeclampsia.

The nomogram approach provides a visual and individualized prediction, which can make it easier for obstetricians to evaluate each pregnant woman’s risk of LBW based on her score and subsequently provide individualized treatment. Furthermore, the nomogram model makes it simple and straightforward for pregnant women who are personally at risk for LBW babies to participate in the intervention and receive superior clinical results. Additionally, this study’s predictive model was developed using the maternal clinical data from early pregnancy. It can be used for screening expectant mothers for LBW at this early stage of pregnancy, allowing for the earlier implementation of effective intervention and treatment for these pregnant women. More significantly, the screening tool’s variables are simple to understand.

Several approaches were used to assess the nomogram’s performance and discrimination. The AUROC value of 0.843 proved that the nomogram had good discrimination power (23). Similarly, models comprising different characteristics of pregnant women presented comparable ability, which achieved an AUC of 0.83, which is nearly the same with our study (11). This comparable discrimination ability of these models might be similar in predictors used for the model development.

This study conducted for predicting LBW in India revealed an AUC value of 0.762 (28). Similarly, a nomogram having seven predictors in China, including the mother’s education level, prenatal care, the mother’s work, pregnancy-induced hypertension, family income, pesticide exposure, and nutritional supplements, could gain an AUC of 0.698 (5). In another study conducted in Uganda, predictors of LBW include gravidity, educational level, serum Alanine Transaminase (ALT), serum Gamma-Glutamyl Transferase (GGT), lymphocyte count, placental position, and end-diastolic notch in the uterine arteries, all of which are factors to consider for an AUC of 81.9% to predict LBW (29). Our model has higher predictive performance compared to previous studies, possibly because the predictors used in our model development had a smaller effect on the risk discriminating ability. However, the predictors they used for model development mostly included advanced laboratory and imaging, which are not routinely available, not easily accessible, need additional investigation, and are difficult to apply in our setting. This renders those models as less practical.

The developed model was well calibrated, showing its reliability (consistency between predicted and observed values), and it revealed similar discrimination performance after internal validation, indicating that it is capable of predicting LBW in an independent set of pregnant women with a comparable degree of accuracy. The decision curve analysis showed that the established model for early risk classification of pregnant women for LBW has a higher clinical benefit than treating none and treating all pregnant women across a wide range of threshold probability. The concept of decision curve analysis involves the standard net benefit along the threshold probability, which is determined by the difference between benefit and cost. In this study, the benefit refers to treating true positives (after correctly predicting a pregnant woman will have an LBW baby) while the cost is treating false positives (after incorrectly predicting a pregnant woman will have an LBW baby).

### Strengths and limitations of the study

First, the study was conducted with a sufficient number of participant outcomes for LBW prediction. This helped in building the model that has an adequate number of predictor variables while preventing overfitting. Second, we have developed a visual diagnostic tool for LBW that enables patients and physicians to make individualized predictions quickly and easily. Additionally, local governments and healthcare providers can use our prediction tool as a guide to enhance pregnancy outcomes.

The study was not without limitations; it would have been better if it had been conducted using a prospective follow-up study design. In retrospectively collected data, some variables that predict LBW might have been missed. However, the nomogram developed using retrospectively collected data are still important in resource-limited settings, including Ethiopia. Additionally, the model was not externally validated using an independent dataset. It would have been better if it had gone through external validation to ensure its prediction capability when applied to other contexts.

In conclusion, this study developed and validated a clinical nomogram for the prediction of LBW risk in Ethiopia. In areas with no laboratory tests and ultrasound scans, a woman’s maternal history can be used to determine the risk of a LBW baby. Factors such as being under the age of 20, having hemoglobin levels less than 11 mg/dL, being a primigravida, having an unplanned pregnancy, and experiencing preeclampsia are all independent risk predictors of LBW. We found that the discrimination ability of this nomogram is highly valuable and can provide significant clinical and public health benefits. We recommend clinicians to utilize this nomogram using the appropriate cutoff point provided to categorize pregnant women. For researchers validating the prediction tool in another context, this would also be a useful approach.

## Data availability statement

The original contributions presented in the study are included in the article/supplementary material, further inquiries can be directed to the corresponding author.

## Ethics statement

The studies involving humans were approved by Institutional Review Boards (IRB) of the University of Gondar. The studies were conducted in accordance with the local legislation and institutional requirements. Written informed consent for participation was not required from the participants or the participants' legal guardians/next of kin because We used secondary data. We only extracted clinically relevant information, did not affect the treatment and health of patients, and the patients' privacy was protected, so written informed consent was not applicable for this study.

## Author contributions

BF: Conceptualization, Data curation, Formal analysis, Investigation, Methodology, Project administration, Resources, Software, Supervision, Validation, Visualization, Writing – original draft, Writing – review & editing. GT: Data curation, Formal analysis, Software, Supervision, Validation, Writing – review & editing. TG: Supervision, Validation, Visualization, Writing – review & editing. MA: Software, Software, Validation, Visualization, Writing – review & editing.
